# Ceramic Membranes Photocatalytically Functionalized on the Permeate Side and Their Application to Water Treatment

**DOI:** 10.3390/membranes9050064

**Published:** 2019-05-23

**Authors:** André Ayral

**Affiliations:** Institut Européen des Membranes, CNRS/ENSCM/Université de Montpellier, CC047, 2, place Eugène Bataillon, 34095 Montpellier CEDEX 5, France; andre.ayral@umontpellier.fr

**Keywords:** ceramic membranes, photocatalysis, hybrid process, permeate treatment

## Abstract

This work deals with direct coupling of membrane separation and photocatalytic degradation by using photocatalytic ceramic membranes. An unusual configuration is considered here, with the irradiation applied on the permeate side of the membrane in order to mineralize small organic molecules not retained by the membrane. Different types of such membranes are presented. Their functional performance is quantified thanks to a simple experimental method enabling the estimation of the specific degradation rate *δ*, i.e., the quantity of destroyed organic molecules per unit of time and of membrane surface area. The relevance of *δ* for the design and scale-up of purification units is then illustrated. Finally, current technological challenges and potential solutions concerning the industrial implementation of such photocatalytic membranes are discussed.

## 1. Introduction

Fifteen years ago, based on our expertise in titania ceramic membranes and considering the growing number of papers dealing with photocatalysis and its applications to the environment protection, we decided to open a new research topic on titania-based photocatalytic membranes. The literature review highlighted several pioneering researches [1,2,3,4,5,6,7,8]. So, we decided to focus our efforts on an unusual configuration, with the irradiation applied on the permeate side of the membrane.

For this short paper, submitted in this Special Issue, the objective is not to propose an additional review article on the subject but to present a compilation of different studies, carried out by our team for several years, on the development and on the characterization of photocatalytic ceramic membranes adopting this unusual configuration.

Brief reminders on photocatalysis principles and on hybrid processes coupling photocatalysis and membrane separation are presented first. Different ceramic membranes, photocatalytically functionalized by us on the permeate side, are then presented. A simple methodology for measuring the functional performance of such membranes is described. Its pertinence for sizing and parameterizing a technological device is tested. The last part of the article deals with current technological challenges and potential solutions for a large-scale implementation of these photocatalytic ceramic membranes.

## 2. Background on Photocatalysis and Hybrid Processes Coupling Photocatalysis and Membrane Separation

Heterogeneous photocatalysis are associated with a complex set of reactions occurring on the surface of solid catalysts, usually semiconducting materials. Their photoexcitation requires their irradiation with a light wavelength lower than their band gap [9,10]. After charge separation with holes h^+^ in the valence band and electrons, e^−^, in the conduction band, the holes, h^+^, can react with electron donors like adsorbed water or hydroxyl ions. The resulting radicals, OH^•^, are powerful oxidizing species enabling a complete mineralization of organics. As a consequence, heterogeneous photocatalysis appears as a promising advanced oxidation process for technological applications in the depollution of air [11] or water [12]. However, it seems restricted to applications with low flows and low pollutant concentrations [13].

Two semi-conducting single oxides, mainly TiO_2_ but also ZnO, are known for their photocatalytic activity under near ultraviolet (UV) irradiation [9]. Concerning titania, its two main crystalline forms, anatase and rutile, are photoactive, with band gap values equal to 3.23 eV (384 nm) and 3.02 eV (411 nm), respectively [14]. Anatase is known as the most photoactive and is used as a reference material in terms of photocatalysis performance. However, mixtures of anatase and rutile phases can exhibit better performance than pure anatase [15,16]. In addition, crystallite sizes around 8 nm offer a good compromise [17,18,19]. Smaller crystallites favor electron-hole recombination and larger ones offer a smaller photoactive surface area. The zinc oxide band gap is also located in the near UV range, with a value of 3.33 eV (372 nm) [20].

Hybrid processes coupling membrane separation and photocatalysis have been investigated since the end of the 1990s [1,2,3,4,5,6,7,8]. The investigated applications concerned, essentially, water treatment, with processes mainly operating with TiO_2_ particles in suspension or supported on substrates, and not with photocatalytic membranes [3,6]. Only few papers or patents describe photoactive membranes with titania nanoparticles embedded in a polymeric membrane for antifouling or waste water treatment [1,4,5,8] or photoactive TiO_2_ membranes for Volatile Organic Compound (VOC) removal [2,7].

More and more papers dealing with photocatalytic titania-based membranes have been published since these pioneering studies. We can here mention the contributions of Choi et al. on anti-fouling and disinfecting uses of titania-based membranes [21,22]. Exhaustive reviews on photocatalytic membrane reactors (PMRs) are also now available [23,24].

## 3. Direct Coupling Considering an Unusual Configuration of Photocatalytic Membrane

In direct coupling of membrane separation and photocatalysis by implementing a photocatalytic membrane, different configurations can be distinguished [23]. The most common design, and the most investigated one, corresponds to a photoactive separative top-layer deposited on a porous substrate. UV irradiation is performed on this multifunctional layer in contact with the feed solution. The main targeted applications concern anti-fouling and photocatalytic reactors.

**Figure 1:**
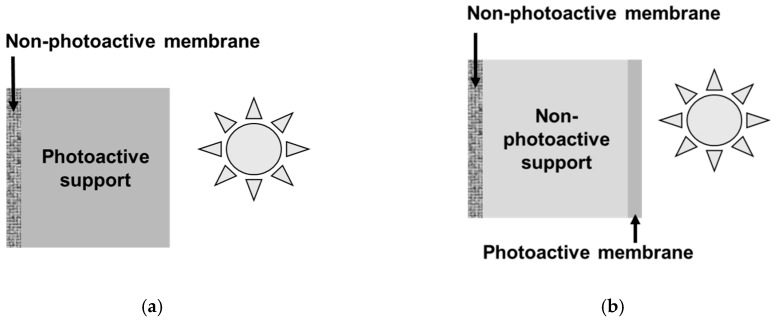
Photocatalytic membranes with ultraviolet (UV) irradiation on the permeate side; (**a**) intrinsically photoactive substrate, and (**b**) covering of the substrate grains with a photocatalytic coating or photoactive film deposited on the porous substrate.

The second and less common configuration corresponds to the case of an asymmetric membrane photocatalytically functionalized on the permeate side. It could be possible thanks to a porous substrate with intrinsic photocatalytic properties (Figure 1a). However, considering the limited penetration depth of UV light in photocatalytic films (no more than few µm) [25], more attractive options are the covering of the grains forming the porous substrate with a photocatalytic phase or the deposition of a thin photoactive film on the porous substrate (Figure 1b).

The interest of such a configuration can be illustrated by the case of wastewater treatment using a low ultrafiltration membrane, retaining nanometer-sized species (colloidal nanoparticles or macromolecules) on the feed side, and photodegrading organic micropollutants on the permeate side.

Until now, very few papers dealt with photocatalytic functionalization of a membrane on the permeate side. They were mainly published by us [26,27,28,29], but also by Romanos et al. [30,31,32,33,34], Guo et al. [35], and Horovitz et al. [36,37].

Concerning the type of membranes used, the main issue with composite membranes, based on organic matrix and embedded photocatalytic nanoparticles, is about durability due to the progressive photodegradation of the organic matrix under irradiation and to the associated progressive release of the photoactive nanoparticles. On this basis and considering our know-how in ceramic membranes, we focused our efforts on the development of photocatalytic ceramic-based membranes.

In the frame of successive research projects completed in our group, such membranes were prepared using various sol-gel routes and also plasma-enhanced chemical vapor deposition (PECVD). For evaluating and comparing their photocatalytic performance, experiments were carried out, in particular, using the same type of porous alumina disks as substrates (diameter 47 mm, thickness 1 mm, from Inocermic).

**Figure 2:**
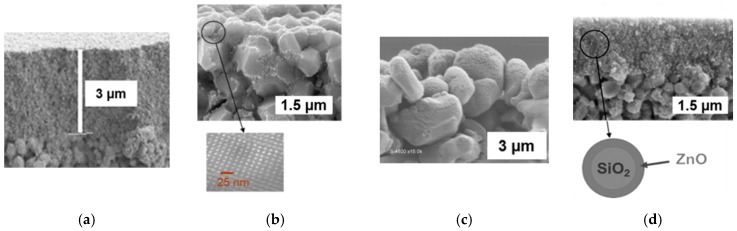
Cross section images obtained by a scanning electron microscope. (**a**) Anatase film from a commercial titania hydrosol, (**b**) coating on substrate grains from a nanocrystalline titania sol, and, as an insert, a transmission electron microscope image showing the ordered mesoporosity of the coating, (**c**) PECVD titania coating on substrate grains, and (**d**) mesoporous film of SiO_2_/ZnO nanoparticles.

For preparing titania-based membranes, three routes were considered and here are briefly described. If required, more details are available in the referenced papers. A simple and robust route used a commercial anatase hydrosol (S5-300B from Millennium Inorganic Chemicals France) [28]. Mesoporous thin films were dip-coated on alumina disks with a 200 nm pore-sized top layer (Figure 2a). An original method was also developed for preparing nanocrystalline anatase hydrosols and resulting coatings with an ordered mesoporosity [26,38,39]. Figure 2b shows substrate grains covered with such a mesostructured anatase coating. In that case, the used alumina substrates had a 200 nm pore-sized top layer. Another pathway was considered for covering the substrate grains with an anatase coating. It was done by PECVD, using titanium tetra-isopropoxide as a TiO_2_ precursor, with a thermal post-treatment for anatase crystallization [29,40,41]. Grains of alumina disks with an 800 nm pore-sized top layer, covered with such a PECVD coating, are shown in Figure 2c.

Due to the unavailability of methods for access to stable concentrated ZnO sols, an original route was developed consisting of the covering of SiO_2_ nanoparticles with a ZnO shell, starting from a commercial silica hydrosol [27]. From such SiO_2_/ZnO core-shell nanoparticles, mesoporous films were dip-coated on alumina disks with a 200 nm pore-sized top layer. A cross-section image of such a film, post-treated up to 500 °C, is shown in Figure 2d.

## 4. A Simple Method for Estimating the Performance of Photocatalytic Membranes

**Figure 3:**
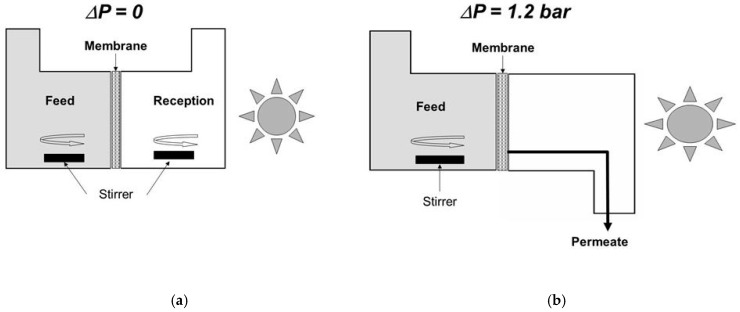
Schematic representations of cells used for assessing the functional performance of membranes photocatalytically functionalized on the permeate side. (**a**) Diffusion set-up and (**b**) permeation set-up.

We developed a simple method for assessing the operational performance of the various membranes photocatalytically functionalized on the permeate side. It is based on a diffusion cell with two compartments, separated by such a functionalized ceramic plate (Figure 3a) [26]. The feed compartment is filled with an aqueous solution of organic dye, namely Methylene Blue (MB), or of a low molecular weight organic compound, namely phenol. All the photocatalysis experiments described in this section were done using the same UV lamp with a polychromatic spectrum exhibiting a maximum around 350 nm and with a same irradiance of 35 W·m^−2^, measured with a UV radiometer at the level of the membrane surface in the absence of liquid. In comparison with the total diameter of the used disks (47 mm), the effective one was equal to 40 mm, corresponding to an irradiated area of ~12.6 cm^2^. Under standard conditions, the feed compartment contained an aqueous solution with a MB concentration of 10^−4^ mol·L^−1^ or a phenol concentration of 10^−3^ mol·L^−1^. The reception compartment was initially filled with pure water. After a contact time of one day, in order to saturate the membrane with adsorbed organic molecules, the feed and reception compartments were again filled with the aqueous solution and with pure water, respectively. This operation corresponds to the beginning of the diffusion experiment.

**Figure 4:**
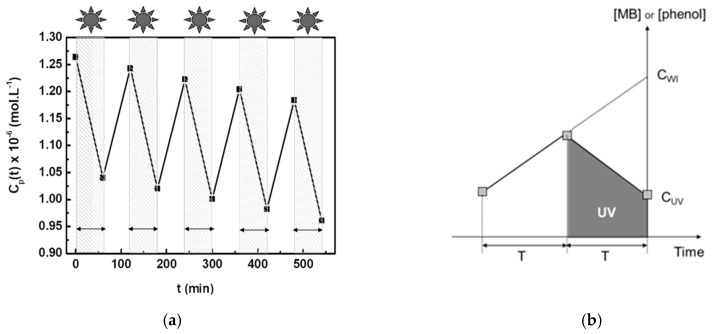
Measurement of the parameter *δ* equal to the quantity of organic solute photodegraded per units of time and of membrane surface area. (**a**) Concentration of Methylene Blue (MB) in the reception compartment with alternate periods with or without UV irradiation, using the macroporous disk with grains coated by mesostructured anatase. (**b**) Graphical representation of the method used for calculating *δ*.

UV irradiation is alternately (usually periods of one hour) applied on the reception side, inducing concentration variations in this compartment (Figure 4a). During the periods without irradiation, the concentration of the organic compound increases due to the diffusive flux of solute from the feed compartment (initial MB concentration of 10^−4^ mol·L^−1^ or initial phenol concentration of 10^−3^ mol·L^−1^) to the reception initially filled with pure water (initial solute concentration equal to 0). During the periods with irradiation, the solute concentration in the reception compartment decreases because the amount of solute instantaneously photodegraded is larger than that simultaneously arriving in this compartment due to the diffusion phenomenon. It is then possible to determine the specific degradation rate, *δ*, equal to the quantity of organic solute photodegraded per unit of time and of membrane surface area.

Figure 4b shows a graphical representation of the calculation method for *δ*. The concentration of organic solute, *C_UV_*, is measured in the reception compartment at the end of an irradiation period of time *T*. The concentration of the organic solute, which should be measured in the absence of irradiation, *C_WI_*, is determined from the linear extrapolation of the concentration increase during the previous period of time without UV irradiation, *T*. The volume of liquid in the reception compartment and the membrane area are named *V* and *A*, respectively. The value *δ* is given by the following equation:

*δ* = *V* × (*C_WI_* − *C_UV_*)/(*T* × *A*)

The specific degradation rate, *δ*, has been quantified for all the membranes shown in Figure 2. The corresponding values are grouped in Table 1. The macroporous substrate, with grains coated by mesostructured anatase, with high photocatalytic activity [38] gives the highest value of *δ*. The value *δ* is five to ten times smaller for substrates covered by an anatase film deposited from the commercial titania sol and for substrates with grains coated by a PECVD layers. For the ZnO-based film, *δ* is one order of magnitude smaller. This ranking is in qualitative agreement with that resulting from experiments of MB photodegradation in batch conditions using suspensions of equivalent powders.

The overall efficiency of these photocatalytic contactors can be discussed considering the elemental photocatalytic microreactors corresponding to the individual pores opened at the permeate surface, the surface of which is coated with photocatalyst and is irradiated by UV light [29]. The residence time of the solute in these elemental microreactors is a complex function of the permeate flow, of the solute self-diffusion in the liquid phase, and of the pore size and geometry. The intrinsic efficiency of the photocatalyst is also very important because it defines the photodegradation rate for the solute molecules arriving on the photoactive surface.

Smaller pore sizes for the porous medium crossed by the permeate should increase the probability for a solute molecule to be in contact with the photoactive walls and, thus, to be degraded. The data reported in Table 1 clearly show that this confinement effect does not play a major role in the overall performance quantified by *δ*. As a matter of fact, the membrane based on the mesostructured anatase coating on substrate grains, with a mean pore size of 200 nm, is five times more efficient than the mesoporous film of anatase with a mean pore size of 11 nm. On the other hand, the thicknesses of the functionalized layers (Table 1) are all greater than or equal to the penetration depth of the UV light inside. By way of consequence, all these membranes cannot be differentiated by their irradiation depth, which is expected to be more or less the same for all of them. As previously mentioned, for anatase, a crystallite size around 8 nm offers the best compromise between the electron-hole recombination rate and good accessibility to the crystallites surface. From the available data concerning the crystallite sizes (Table 1), it is again not possible to explain the observed differences of *δ* between the three titania-based membranes. They can however be differentiated by their nanostructure, which controls the accessibility to the active surface. When available, the values of specific surface area, mean pore size, and porosity of the photocatalytic phases have been reported in Table 1. The mesostructured anatase coating exhibiting the largest surface area and ordered mesopores, expected to be more accessible for the solute molecules, is actually that exhibiting the highest value of *δ*.

Numerical simulation using model microreactors would undoubtedly be useful for a better understanding of all these experimental results and also for optimizing the design of the photocatalytically functionalized membranes.

## 5. Implementation in Representative Conditions and Design of Purification Units

As an initial approach, the previously determined specific degradation rate, *δ*, can be used for assessing the applicability of such photocatalytic membranes considering the pollutant content and flow of water to be treated. It enables a preliminary estimate of the size and working conditions of purification units to be implemented.

**Figure 5:**
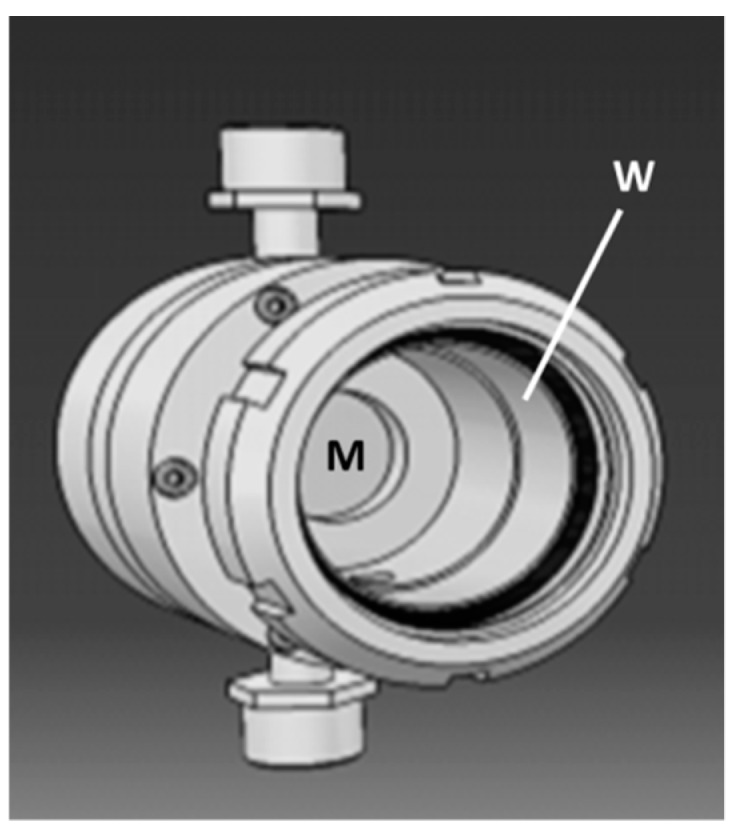
Membrane module especially designed for coupling photocatalysis and membrane filtration. (**M**) flat membrane; (**W**) window in borosilicate glass transparent to near-UV light.

Experiments carried out by applying a transmembrane pressure for inducing transmembrane liquid flow (Figure 3b) will now be considered. A homemade membrane module (Figure 5) was especially designed for coupling photocatalysis and membrane filtration with a macroporous ceramic disk [42]. In comparison with the total diameter of the used disks (47 mm), the effective one was equal to 36 mm, corresponding to an irradiated area of ~10.2 cm^2^. Its functionalization on the permeate side was done by the deposition of an anatase film prepared from the commercial sol (Figure 2a). This filtration module was placed in the loop of a membrane pilot [42]. The circulation speed in the pilot loop was equal to 2.7 m·s^−1^. An aqueous solution of phenol with a concentration equal to ~10^−5^ mol·L^−1^ was used as feed solution. For a transmembrane pressure, Δ*P*, of 1.2 bar, the measured permeance *P_e_* was equal to ~40 L·h^−1^·m^−2^·bar^−1^ = ~0.011 L·s^−1^·m^−2^·bar^−1^. Considering a value of *δ* for phenol equal to 2 10^−8^ mol·s^−1^·m^−2^, the expected decrease of phenol concentration in the permeate was thus equal to (*P_e_* × Δ*P*/*δ*) = 1.5 10^−6^ mol·L^−1^.

Due to the low transmembrane pressure applied, the experiments were performed without a glass window in order to maximize the light irradiation of the membrane surface on the permeate side. Two types of experiments were carried out. The first ones were done with the membrane module placed vertically and with or without irradiation by a UV lamp with an irradiance of 35 W·m^−2^. For the second ones, performed outside, the module was inclined at 45° upward and oriented in a southern direction. These experiments were done during a sunny day with a measured irradiance of 45 W·m^−2^ for the solar radiation. The concentrations of phenol in the permeate for different experimental conditions are reported in Table 2. It can be first noted that, despite a molecular weight cut-off of ~50 kDa previously measured for the tested membrane [28], the retention of phenol (94 Da) in absence of irradiation is quite important (*C_PV_* and *C_PI_* are significantly smaller than *C*_0_). This phenomenon could be due to phenol adsorption on the pore surface in the membrane. Apart from this, the reported data clearly show that the decrease of phenol concentration measured in the permeate during the UV or solar irradiation periods, (*C_PV_* − *C_UV_*) and (*C_PI_* − *C_SI_*), are in the same order of magnitude as that expected from the value of *δ* determined in static conditions (using the diffusion cell), (*P_e_* × Δ*P*/*δ*).

Such agreement between the expected decrease of pollutant concentration in the permeate calculated from *δ* and the measured change in dynamic condition, i.e., by applying a transmembrane pressure, was also verified in the case of porous ceramic discs functionalized on the permeate side with a PECVD anatase coating (Figure 2c) [29].

A new homemade pilot, including four single-channel tubular membranes all simultaneously irradiated on their external side over a length of 160 mm, has been built and will soon be implemented for additional validation at a larger scale and with both model and real wastewaters.

## 6. Current Technological Challenges and Potential Solutions

Integration and process intensification are two important challenges concerning the technological implementation of photocatalytic membranes.

**Figure 6:**
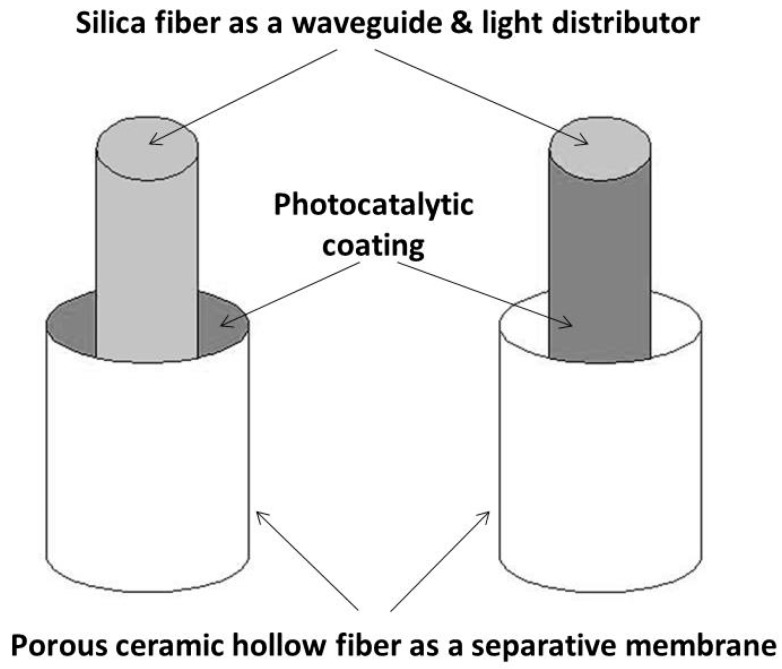
Possible configurations for coupling ceramic hollow fibers used as separation membranes and optical fibers used as light distributors.

The design of compact modules with a large surface area of membrane is required. Optical fiber tissues have been recently developed [43]. They could be used as spacers and light distributors in spiral modules of photocatalytically functionalized organic membranes. On the other hand, the long-term degradation of the organic membranes under UV irradiation can be expected. Such drawback can be avoided using ceramic membranes. However, due their stiffness and brittleness, spiral modules cannot be obtained with ceramic membranes. One possible option would be the use of ceramic hollow fibers and of individual optical fibers as light distributors (Figure 6) [44]. Previous studies showed the feasibility of compact photocatalytic reactors using single optical fibers [45,46]. However, the manufacturing of such modules seems complex.

In terms of process intensification, new low-energy consumption UV LEDs have now to be considered as irradiation sources. The use of solar radiation (discontinuously available) with photocatalysts exhibiting a band gap in the visible range is another option. Titania doping enables a band gap shift in the visible range [36,37,47]. Impressive efforts are also currently done for developing new photocatalysts highly active in the visible range.

Except for industrial wastewaters, another important issue is the variability in terms of chemical composition for the water to be treated. Photocatalysis does not systematically give rise to a full mineralization. Products resulting from incomplete photodegradation can be more toxic than the starting solutes. As a consequence, on-line systems enabling continuous chemical analysis of feed and permeate should be integrated for the optimized monitoring of advanced technological facilities integrating photocatalytic membranes.

## 7. Conclusions

For twenty years, an increasing interest has been paid to hybrid processes coupling membrane separation and photocatalysis, especially by using photocatalytic membranes. Our own efforts have been focused on photocatalytic ceramic membranes considering a rather unusual configuration corresponding to the functionalization of the membrane on the permeate side. In addition to its separation function, the membrane is used as a contactor for the photodegradation of small species not retained by the membrane.

In this frame, a simple test has been developed using a diffusion cell, which leads to the determination of the specific degradation rate *δ*, an operational parameter equal to the quantity of destroyed organic solute per units of time and of the membrane surface area. The value *δ* enables the estimation of the size and working conditions of the purification units to be implemented.

The main remaining challenges for technological applications of photocatalytic membranes are related to integration issues with the design of compact modules with high membrane surface areas and to process intensification with the reduction of the energy cost associated with the required irradiation of the functionalized surfaces.

## Figures and Tables

**Table 1:** Measured values of δ and other features of all the membranes shown in Figure 2.

Tested Membrane	TiO_2_ film from Commercial Hydrosol (Figure 2a)	Mesostructured TiO_2_ Coating on Substrate Grains (Figure 2b)	PE-CVD TiO_2_ Coating on Substrate Grains (Figure 2c)	Film of SiO_2_/ZnO Particles (Figure 2d)
*δ* for MB (mol·s^−1^·m^−2^)	(2.0 ± 0.5) × 10^−8^	(1.0 ± 0.2) × 10^−7^	(1.5 ± 0.5) × 10^−8^	(1.5 ± 0.5) × 10^−9^
*δ* for phenol (mol·s^−1^·m^−2^)	(3 ± 1) × 10^−8^	-	-	-
Mean pore size of the photoactive layer (nm)	11*	200	800	6*
Thickness of photoactive layer (µm)	~3	>>10 infiltration in the substrate	<5	~1.5
Crystallite size of the photocatalytic phase (nm)	5–10	8	20	10
Specific surface area of the photocatalytic phase (m^2^·g^−1^)	143*	190*	-	36*
Mean pore size of the photocatalytic phase (nm)	11*	4.2*	-	6*
Porosity of the photocatalytic phase (%)	65*	40*	33	15*

* Data from powders obtained after drying and thermal treatment of the starting suspensions in the same conditions as for the deposits.

**Table 2:** Phenol concentrations (expressed in 10^−6^ mol·L^−1^): in the feed solution, *C*_0_; in the permeate without irradiation and with the module in vertical position, *C_PV_*; in the permeate with UV irradiation and with the module in vertical position, *C_UV_*; in the permeate without irradiation and with the module inclined at 45° upward, *C_PI_*; and in the permeate with solar irradiation and with the module inclined at 45° upward, *C_SI_*.

**Concentration** **(10^−6^ mol·L^−1^)**	*C*_0_	*C_PV_*	*C_UV_*	*C_PV_* − *C_UV_*	*C_PI_*	*C_SI_*	*C_PI_* − *C_SI_*
	9.8 ± 0.2	7.4 ± 0.2	5.7 ± 0.2	1.7 ± 0.4	8.2 ± 0.4	6.2 ± 0.2	2.0 ± 0.6
